# Multimedia Transmission over LoRa Networks for IoT Applications: A Survey of Strategies, Deployments, and Open Challenges

**DOI:** 10.3390/s25237128

**Published:** 2025-11-21

**Authors:** Soumadeep De, Harikrishnan Muraleedharan Jalajamony, Santhosh Adhinarayanan, Santosh Joshi, Himanshu Upadhyay, Renny Fernandez

**Affiliations:** 1 Department of Materials Science and Engineering, Norfolk State University, Norfolk, VA 23504, USA; s.de@spartans.nsu.edu (S.D.); h.jalajamony@spartans.nsu.edu (H.M.J.); 2 Department of Electronics Engineering, Norfolk State University, Norfolk, VA 23504, USA; s.adhinarayanan@spartans.nsu.edu; 3 Department of Electrical and Computer Engineering, Florida International University, Miami, FL 33199, USA; sajoshi@fiu.edu (S.J.); upadhyay@fiu.edu (H.U.)

**Keywords:** LoRa, LPWAN, image transmission, low-power wireless, compression, MAC protocols, cooperative communication, wireless sensor networks, edge computing, smart agriculture, environment monitoring, surveillance systems, IoT

## Abstract

LoRa has emerged as a cornerstone of low-power, long-range IoT communication. While highly effective for scalar sensing, its extension to multimedia remains constrained by limited bitrate, payload size, and duty-cycle regulations. This survey reviews research on multimedia transmission over LoRa, revealing that most current efforts are image-centric, with only a few preliminary studies addressing video or audio. We propose a structured taxonomy encompassing compression and fragmentation methods, cooperative and multi-hop architectures, MAC and cross-layer optimizations, and hybrid network designs. These strategies are analyzed in the context of IoT domains such as agriculture, surveillance, and environmental monitoring. Open challenges are highlighted in extending beyond static images, ensuring energy-efficient delivery, and developing spectrum- and ML-aware protocols. The survey provides IoT researchers with both a consolidated reference and a roadmap toward practical and scalable multimedia systems over LoRa.

## 1. Introduction

The proliferation of Internet of Things (IoT) applications has driven demand for low-power, long-range communication technologies. Among these, LoRa (Long Range) has gained significant traction due to its ability to transmit small packets over several kilometers using minimal energy. While originally intended for scalar sensor data, LoRa’s use is expanding to include multimedia applications such as remote surveillance [1,2], agricultural monitoring [3,4,5,6,7,8,9], environmental monitoring [10,11,12], and wildlife tracking [13,14,15,16], where image and video data are increasingly relevant.

However, transmitting multimedia content over LoRa presents substantial challenges. LoRa operates at low bitrates, reaching a maximum of approximately 35 kbps under optimal settings, and supports payload sizes of only up to 255 bytes per packet [17,18]. In addition, unlicensed band regulations such as FCC dwell-time restrictions and 1% duty-cycle limits [19,20] significantly constrain how frequently devices can transmit. These limitations are especially problematic for image or video transmission, which demand low latency, high throughput, and loss resilience. To address this, researchers have developed a variety of strategies across the protocol stack, including compression schemes, dual-radio and SF-diverse transmission, cooperative relays, adaptive MAC protocols, and hybrid LoRa + WSN architectures.

To ensure comprehensive coverage, relevant works were identified through a systematic search across major scientific databases, including IEEE Xplore, ScienceDirect, SpringerLink, MDPI, and ACM Digital Library. The search employed keywords such as “LoRa image transmission,” “LoRa multimedia,” “LoRa video,” “LoRa sensing network,” and “multimedia over LPWAN.”

A notable early effort to consolidate this research landscape was presented by Staikopoulos et al. [21], who reviewed foundational techniques including JPEG and JPEG2000 compression, CSMA-based access, patch-based transmission, and Multi-Packet LoRa (MPLR). Their work identified key constraints posed by LoRa’s bandwidth and regulatory restrictions and emphasized the need for both image and protocol-level optimization. However, their survey predated recent advances in real-world deployments, concurrent reception, frequency division forwarding, Machine Learning (ML) aware compression, and robust hybrid designs. Furthermore, it lacked a structured taxonomy or comparative analysis of systems, performance metrics, or domain-specific adaptations.

This survey addresses those gaps by providing the most comprehensive and technically grounded review to date of image and video transmission over LoRa networks. We introduce a structured taxonomy to classify existing systems into three main categories: (1) compression strategies tailored to LoRa’s rate and payload constraints, (2) cooperative and multi-hop transmission architectures that extend range and reduce latency, and (3) MAC and protocol-level enhancements designed to optimize throughput, energy usage, and fairness. In addition, we analyze a wide range of real-world deployments spanning smart agriculture, surveillance, and environmental sensing, evaluating how each system applies core strategies under practical constraints. A dedicated section discusses open research problems, such as scalable cross-layer coordination, ML-aware compression for inference tasks, energy-adaptive scheduling, and frequency-diverse packet planning.

This paper makes the following contributions:

- We provide a structured taxonomy of LoRa-based multimedia transmission strategies, including compression, cooperative forwarding, and MAC-layer optimization.
- We synthesize insights from a broad set of peer-reviewed works, analyzing performance metrics, hardware platforms, and deployment settings through unified tables and thematic summaries.
- We identify unresolved challenges and propose open research directions toward scalable, energy-efficient, and ML-integrated image transfer frameworks over LoRa.

While broadband IoT technologies such as NB-IoT and RedCap can support higher data rates, their higher cost, energy consumption, and dependence on terrestrial infrastructure limit their suitability for many remote or power-constrained scenarios. In contrast, LoRa offers a practical solution for sparse terrestrial deployments and bandwidth-limited environments, where long-range, low-power communication is prioritized over throughput. This also extends to emerging satellite-based LoRa links operating within the constrained L-band spectrum, which enable image or event monitoring in areas lacking conventional network coverage.

The rest of the paper is organized as follows. Section 2 reviews the physical and regulatory limitations of LoRa for multimedia data. Section 3 introduces the proposed taxonomy. Section 4, Section 5 and Section 6 analyze compression techniques, cooperative transmission systems, and MAC/protocol optimizations, respectively. Section 7 summarizes real-world deployments by domain. Section 8 outlines open research problems. Section 9 concludes the paper.

## 2. Background on LoRa and Multimedia Constraints

### 2.1. LoRa Physical Layer and Protocol Characteristics

LoRa is a proprietary physical layer modulation scheme developed by Semtech, based on Chirp Spread Spectrum (CSS) [22,23,24,25]. It operates in unlicensed sub-GHz ISM bands such as 868 MHz (EU), 915 MHz (US), and 433 MHz (Asia), offering communication over several kilometers with ultra-low power consumption. These characteristics have made LoRa a foundational technology for long-range, battery-operated sensor networks [26,27,28,29].

Three key configurable parameters determine the trade-off between data rate, range, and energy usage: Spreading Factor (SF), Bandwidth (BW), and Coding Rate (CR) [30,31,32,33,34,35]. The SF, ranging from 6 to 12, controls the symbol duration, with higher values improving sensitivity at the cost of lower throughput. The BW, commonly set to 125 kHz, 250 kHz, or 500 kHz, defines the frequency range over which chirps are spread. The CR (typically 4/5 to 4/8) adds forward error correction bits, improving robustness at the expense of payload efficiency [17,18].

The achievable data rate in LoRa varies from approximately 0.3 kbps (SF12, BW = 125 kHz) to a practical maximum of 35 kbps (SF6, BW = 500 kHz, payload = 240 bytes), based on empirical Time-on-Air (ToA) calculations using SX1276 hardware [17,18,28]. While the theoretical PHY limit is higher, actual throughput is constrained by packetization overhead, energy consumption, and regulatory limits, even under high-rate configurations such as SF7 with 500 kHz [36].

LoRa supports a maximum payload size of 255 bytes per packet at the physical layer [17,18]. However, each transmission is subject to a symbol-duration-based ToA, which increases exponentially with SF. This becomes critical under region-specific transmission regulations. For example, the FCC enforces dwell-time limits in the US, while ETSI imposes a 1% duty cycle limit in Europe [19,20]. These constraints limit how frequently nodes can transmit, directly affecting multimedia performance.

**Figure 1:**
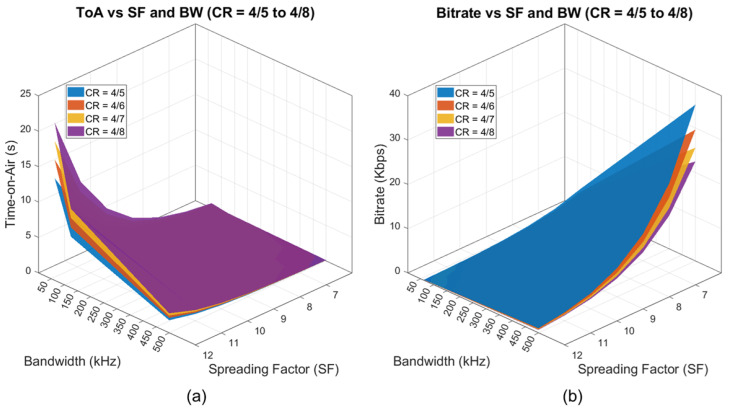
3D surface plots illustrating the impact of Spreading Factor (SF), Bandwidth (BW), and Coding Rate (CR) on (**a**) Time-on-Air and achievable (**b**) Bitrate for a 240-byte LoRa payload. Increasing SF or CR increases ToA, while wider BW reduces it. Bitrate trends inversely with ToA, peaking at SF6 and 500 kHz with CR = 4/5.

The Time-on-Air (ToA) of a LoRa transmission is primarily determined by the Spreading Factor (SF), Bandwidth (BW), and Coding Rate (CR). The symbol duration increases exponentially with SF, while wider bandwidths shorten it [17,18,28]. A higher CR increases the number of bits transmitted per symbol for error correction, further extending ToA. This trade-off is crucial in multimedia transmission, where longer ToA directly impacts latency, energy consumption, and compliance with regulatory duty-cycle constraints. These trends are confirmed in a simulation study by Fialho [37], which models CSS-based image transmission and shows that increasing SF significantly raises ToA while degrading image PSNR under low SNR conditions. Juliando et al. [38] further support this empirically, showing that transmitting a single uncompressed 640 × 320 pixels image using Ra-02 modules at SF12 and 125 kHz results in ToA exceeding 850 min, reinforcing the need for compression and protocol optimization. Figure 1 presents 3D surface plots showing how ToA and bitrate evolve across varying SF, BW, and CR settings for a 240-byte payload. As observed, ToA increases significantly with higher SF values, while lower bandwidth and higher coding rates further extend transmission time. These parameters collectively determine the trade-off between link robustness, achievable throughput, and the likelihood of collisions when multiple devices operate under ALOHA-based channel access (Appendix A.1). Moreover, since only a limited number of discrete SF levels (7–12) are available, multiple terminals using the same SF increase the probability of packet collisions despite the enhanced range and orthogonality offered by higher SF values.

LoRa and LoRaWAN have been extensively deployed in conventional IoT domains such as smart metering, environmental monitoring, precision agriculture, and smart city infrastructure, where low data rate and long-range connectivity are prioritized over bandwidth [39,40,41,42,43,44,45,46]. LoRa is commonly paired with the LoRaWAN MAC protocol, which adds device addressing, acknowledgments, and support for multiple device classes (A, B, C) [47,48,49]. However, many multimedia-oriented systems use customized MAC strategies or operate below the LoRaWAN stack entirely. As such, this survey focuses primarily on physical and MAC-layer techniques that are protocol-agnostic and directly influence image and video transmission feasibility.

### 2.2. Multimedia Transmission Requirements

Transmitting multimedia content, particularly images and video-over low-power wireless links introduces a distinct set of challenges. Even modest-resolution grayscale images (e.g., 200 × 150 pixels) can occupy over 20 kB in JPEG format [50]. Color video frames are significantly larger and require consistent throughput to preserve temporal integrity. These characteristics sharply contrast with LoRa’s design, which targets sporadic, low-volume, delay-tolerant transmissions.

Multimedia applications typically impose three core requirements: (i) high data volume, (ii) latency sensitivity, and (iii) acceptable reconstruction quality [50,51,52]. For instance, transmitting a compressed image of 10 kB may require more than 40 packets under LoRa’s 255-byte payload constraint, and the total airtime can easily exceed several seconds, especially with large spreading factors and regional duty-cycle restrictions. Real-time video applications are even more demanding due to frame deadlines and continuity.

Another significant challenge is fragmentation overhead. Multimedia data must be split into multiple packets, each with sequencing, header, and transmission cost. This adds latency, increases energy consumption, and elevates the risk of packet loss [53,54]. Unlike TCP/IP-based systems, LoRa’s physical layer does not guarantee in-order delivery or retransmission, further complicating reliable reconstruction.

Finally, application tolerance to loss and delay varies. Use cases such as pest monitoring [15] or crop status detection [9] can tolerate lossy or delayed image delivery. In contrast, security surveillance [1,2], environmental monitoring [10,11,12] or wildlife tracking [13,14,15,16] may require near real-time response with high image fidelity. These varying demands motivate the diverse design strategies reviewed in this survey, including optimized compression, adaptive scheduling, and cross-layer coordination.

### 2.3. Summary of Key Constraints

Table 1 summarizes the key constraints impacting multimedia transmission over LoRa networks. These limitations stem from factors at the physical and MAC layers, regulatory restrictions, and application-level demands. A clear understanding of these bottlenecks is critical before assessing the technical strategies presented in the following sections. Figure 1 visually illustrates how spreading factor (SF), bandwidth (BW), and coding rate (CR) influence Time-on-Air and achievable bitrate.

## 3. Taxonomy of Multimedia Transmission Techniques

Multimedia transmission over LoRa is fundamentally constrained by limited bandwidth, strict packet size limits, and duty-cycle regulations. These physical and regulatory barriers, combined with the latency and fidelity demands of image and video data, have motivated a diverse set of engineering responses.

**Figure 2:**
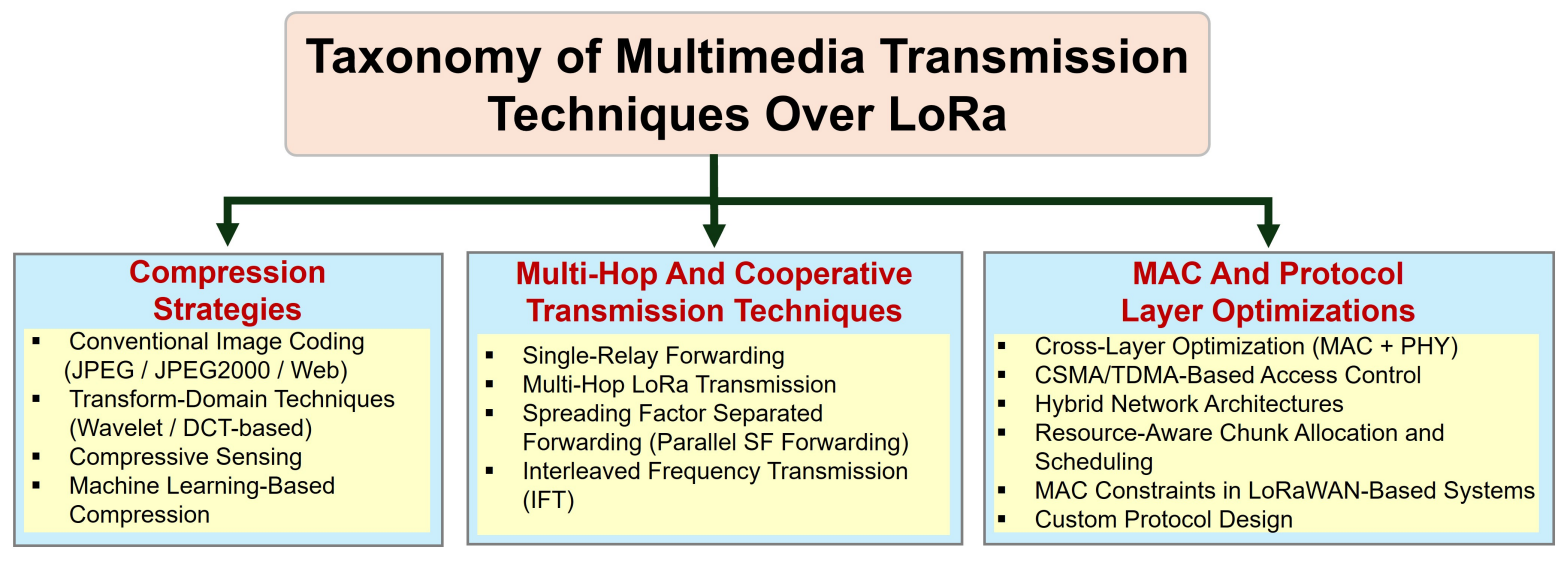
Taxonomy of LoRa-based multimedia transmission strategies.

To bring structure to this design space, we categorize existing techniques into four core strategies, illustrated in Figure 2. These categories also form the organizational backbone of this review:

- **Compression Strategies:** Reducing image size to fit within LoRa’s throughput and airtime constraints. Approaches include JPEG-based encoders, transform-domain methods, and emerging machine learning techniques.
- **Multi-Hop and Cooperative Transmission:** Architectures that split the transmission burden across multiple nodes, frequencies, or radios to reduce latency, increase coverage, or boost throughput.
- **MAC and Protocol Layer Optimizations:** Adjusting medium access (e.g., CSMA, TDMA) and proposing protocol-level innovations (e.g., MPLR, segmentation-aware retransmissions) to improve efficiency and reliability under regulatory and network constraints.
- **Application case Studies:** Real-world deployments that combine the above strategies in domain-specific scenarios such as agriculture, surveillance, and environmental monitoring.

## 4. Compression Strategies

### 4.1. Conventional Image Coding

Kirichek et al. [55] evaluated JPEG and JPEG2000 compression for LoRa-based image and voice transmission using SX1276 radios and STM32L microcontrollers. Experiments over a 5 km UAV-to-ground link showed JPEG2000 offered superior quality with zero MSE and higher PSNR under DR6 (SF7, 250 kHz), while JPEG exhibited up to 18% packet loss under DR4. Subjective video assessments confirmed better visual quality for JPEG2000, with expert ratings averaging 3.24 versus 2.2 for JPEG. These results reinforce JPEG2000’s suitability for high-fidelity multimedia delivery over constrained LoRa links.

Jebril et al. [56] presented an empirical evaluation of JPEG image transmission over LoRa for environmental monitoring in mangrove forests. Using RN2903 transceivers and Arduino-based nodes, the system split encrypted JPEG data into 84-character packets and transmitted them across SF7–SF12. Outdoor field tests demonstrated successful transmission up to 6 km with SF = 12, with PSNR = 24.43 dB and SSIM = 0.9528 reported at optimal configurations. The study highlighted the role of Fresnel zone clearance and spreading factor selection in preserving image fidelity under real-world conditions.

Correia et al. [57] proposed a JPEG-based image acquisition and transmission system using ESP32-CAM and MKR WAN 1310 for LoRaWAN networks. Images (2.6–8.4 kB, 320 × 240 pixels JPEG) were transmitted using a stop-and-wait ARQ protocol with packet sizes of 25–50 bytes and 15–20 s inter-packet intervals. Real-world tests over TTN at distances up to 2.5 km demonstrated reliable delivery under low-frequency, delay-tolerant constraints, with total image transmission times ranging from 24–26 min.

Wei et al. [50,58] investigated the impact of image encoding methods on LoRa transmission efficiency. Using a Raspberry Pi and SX1276 LoRa modules, they compared JPEG, H.264, and WebP with Base64 encoding for a 200 × 150 pixels image over a 1.5 km line-of-sight link. WebP + Base64 produced the smallest file size (3.55 KB) and lowest transmission time (25.7 s), with acceptable quality (PSNR = 33.84 dB, SSIM = 0.904). JPEG required nearly double the time (47.7 s). The results highlight that codec choice significantly impacts performance, and WebP + Base64 is a viable alternative for reducing transmission latency in LoRa-based visual monitoring.

Obeng et al. [52] evaluated JPEG compression and Base64 encoding for LoRa-based image transmission using Raspberry Pi 4 and SX1276 modules. JPEG yielded faster transmission for small to medium images (3–30 KB), while Base64 required more time but preserved image fidelity-provided all packets were received. For large images (164 KB), Base64 failed to reconstruct the image due to error sensitivity, whereas JPEG decoded successfully with some color distortion. The study underscores the trade-offs between compression efficiency, robustness to loss, and visual fidelity under LoRa’s bandwidth constraints.

Zhang et al. [59] proposed a LoRaWAN-based image transmission system using a customized JPEG compression method (CIRAJpeg) and recovery protocol; CIRA (Camera with Image Recovery Algorithm). Targeting QVGA images over Hong Kong’s AS923 LoRaWAN regulation, the system uses headerless JPEG, section-based encoding with pixel position metadata, and packet-aware reconstruction. A loss-resilient preview image is generated using the previous frame, and retransmissions are coordinated via downlinks constrained to TTN (The Things Network) limits. Simulation across four test phases showed CIRAJpeg with recovery outperformed baseline JPEG and headerless JPEG under packet loss, improving visual completeness and transmission feasibility under strict duty cycle constraints.

### 4.2. Transform-Domain Techniques

Guerra et al. [60] proposed a wavelet-based encoder using YCoCg color space, 4:2:0 chroma subsampling, and progressive subband transmission with optional Huffman coding. Evaluated on Lena, Monument, and hydrometric ruler images, the method achieved up to 4% higher compression factor than JPEG2000, with comparable PSNR and SSIM.

Haron et al. [61] applied DCT-based lossy compression using MATLAB R2024b to scale down grayscale images for LoRa-style wireless transmission. Compression was achieved by truncating DCT coefficients, with reported ratios up to 49× for 512 × 512 pixels images at the cost of reduced PSNR (25.75 dB). The method was evaluated on golden apple snail images but lacked hardware validation or integration with LoRa protocols.

### 4.3. Compressive Sensing

Chaparro et al. [62] proposed an image transmission framework using compressive sensing to reduce image size before sending it over LoRa. The system was implemented on Software-Defined Radios (SDR), allowing full control over transmission parameters. A 128 × 128 pixels grayscale image was compressed and transmitted in four packets, with a reported transmission time of 2.51 s and PSNR values up to 30 dB. While the method shows high compression, it relies on complex signal reconstruction and controlled lab conditions. The approach is not yet practical for constrained LoRa devices due to sensitivity to packet loss and the high processing cost of image recovery.

### 4.4. Machine Learning-Based Compression

Sachinda et al. [63] presented an ML-aware image transmission system that aligned JPEG compression levels with neural network robustness to visual degradation. Images originally sized at 60–100 KB were compressed down to under 10 KB using aggressive JPEG quality factors (10–20%) and transmitted via LoRa using ESP32-CAM and SX1278 modules at 433 MHz. Despite the severe compression, classification using pretrained models like ResNet50, MobileNet, and CropNet maintained confidence above 90% in most cases. This showed that lossy compression levels unacceptable to humans could still enable accurate ML inference, allowing LoRa-based systems to meet tight duty-cycle limits while supporting edge intelligence.

Körber et al. [64] presented a deep learning-based image compression method using a lightweight Generative Adversarial Network (GAN) encoder designed for microcontrollers. The focus was on reducing memory and compute usage, not on LoRa transmission. At bitrates down to 0.036 bpp, the model achieved competitive perceptual quality while requiring 99% less memory and 97% fewer operations than standard GAN-based compressors.

Shiddiq et al. [65] proposed a CNN-based autoencoder for compressing 80 × 80 pixels images in autonomous electric vehicle systems using LoRa. The model combined Squeeze-and-Excitation blocks, Subpixel Convolution, and Self-Attention for compact latent encoding, followed by Zlib, Huffman, or adaptive quantization. The best configuration achieved PSNR up to 30.5 dB with latent sizes around 728 bytes. Although the pipeline was benchmarked with synthetic LoRa bandwidth constraints, no real LoRa transmission was implemented. The study focused on reconstruction quality and model variations, not deployment.

### 4.5. Comparative Analysis

**Figure 3:**
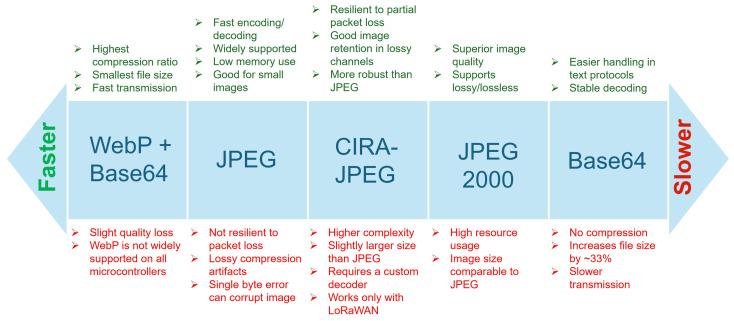
Comparative summary of image compression methods used in LoRa-based multimedia transmission. The figure highlights trade-offs in compression efficiency, transmission speed, error resilience, and hardware compatibility, with methods arranged from faster to slower based on overall transmission performance.

Table 2 and Figure 3 summarizes the key trade-offs across recent compression methods evaluated for LoRa-based multimedia transmission. Techniques differ in compression efficiency, implementation feasibility, and robustness under constrained wireless conditions.

Conventional codecs such as JPEG and JPEG2000 remain the most tested in real deployments. JPEG2000 typically delivers better visual quality at the cost of higher complexity [55], while JPEG is widely used in low-cost embedded systems such as ESP32-CAM. Correia et al. [57] demonstrated a full JPEG-based LoRaWAN system transmitting 320 × 240 pixels images over TTN, with reliable delivery up to 2.5 km. However, the transmission time ranged from 24–26 min per image due to long inter-packet intervals and small payloads.

WebP with Base64 encoding offers reduced transmission time with acceptable quality in low-resolution use cases [50,58], though Base64 introduced size overhead. Transform-domain methods, including wavelet and DCT, achieved moderate gains in compression ratio [60,61] but are mostly evaluated in simulation, without integration into full LoRa stacks.

Compressive sensing (CS) techniques reported high compression under idealized conditions [62], but remain impractical due to reconstruction complexity and loss sensitivity. ML-based compression strategies explored resource-efficient encoders [64] and classifier-aware JPEG tuning [63], but most lack hardware deployment or evaluation over lossy channels.

Overall, conventional JPEG remains the most deployment-ready technique. Transform and ML-based methods show potential for bitrate and fidelity improvements, but rarely meet the constraints of practical LoRa systems.

## 5. Multi-Hop and Cooperative Transmission Techniques

Most cooperative or frequency-division approaches rely on protocol-level relaying or multi-SF forwarding. A complementary direction is hardware-level concurrency, as demonstrated by the Interleaved Frequency Transmission (IFT) method [66]. IFT uses a dual-receiver architecture with two adjacent frequencies (915 and 914.5 MHz), enabling pseudo-parallel reception without altering the LoRa physical layer. Field tests showed a 50% reduction in transmission time and up to 59% energy savings compared to conventional single-frequency setups, highlighting the practicality of dual-radio architectures for accelerating image delivery.

Wei et al. [67] presented a one-relay LoRa image transmission system that uses SF7 for receiving data and SF8 for forwarding it, exploiting spreading factor orthogonality for parallelism. A 240 × 179 pixels JPEG image (10.2 KB) is split into halves and sequentially relayed via two radios connected by MQTT on a Raspberry Pi. This SF-separated approach resembles traditional frequency-offset repeater systems, adapted to LoRa’s modulation constraints.

Tropeano et al. [68] evaluated a multi-hop LoRa system for image transmission using Heltec SX1276 boards in a tree-topology field test. Devices transmitted compressed images (WebP + Base64, 5–20 KB) through up to two hops using D2D relaying with packet-level acknowledgments and retransmissions. Energy profiling showed that intermediate nodes spend most of their time and power in receive mode. Results confirmed that multi-hop relaying extends coverage but introduces latency and energy overheads at relays.

Kim et al. [69] developed and tested a multi-hop LoRa image transmission system using Raspberry Pi boards with RFM95 modules. A custom header based on the RadioHead library supported source, relay, and packet sequencing fields. In field tests, 414–419 byte images were split into 250 byte packets and forwarded across one or two hops. Both single- and dual-sender scenarios were tested, confirming that multi-hop relaying enables image reconstruction at the receiver. Direct transmission was blocked to enforce hop-by-hop delivery, demonstrating basic routing functionality without centralized coordination.

## 6. MAC and Protocol Layer Optimizations

### 6.1. Cross-Layer Optimization (MAC + PHY)

Chen et al. [3] introduced the Multi-Packet LoRa (MPLR) protocol, a cross-layer design for reliable image transmission in LoRa-based IoT systems. MPLR batched packets and used bit-vector acknowledgments (BVACK) to reduce protocol overhead and wait times. Experiments showed an average 24% reduction in image transmission time under ideal conditions, and 30–49% gains under 2–10% packet loss. The authors also proposed a data channel reservation mechanism to prevent collisions in multi-node networks. In star topology tests with 5–20 nodes, MPLR with reservation achieved 2–7× faster delivery and better fairness than stop-and-wait with ALOHA. The core principles of MPLR were later reused in [70,71], though both efforts were limited to single-link implementations without multi-node scheduling or reservation mechanisms.

Wei et al. [72] proposed a parallel LoRa image transmission system that divides a compressed image into segments and distributes them across multiple SF-assigned sender–receiver pairs. Using SF7, SF8, and SF9 simultaneously, each segment is transmitted independently to a corresponding receiver node. MQTT is used for inter-node coordination, and packet reassembly is performed at a central node. Field tests in Taichung City with a 200 × 150 pixels JPEG image show that the 3-to-3 SF-multiplexed setup reduces transmission time from 48 s (1-to-1) to 26 s, without degrading PSNR or PRR.

While Wei et al. distribute SF-separated transmission across multiple nodes, Keshmiri et al. [73] centralized the process within a dual-radio end device. A dual-radio node integrates two synchronized SX1276 transceivers, each operating on a different SF (e.g., SF7 with SF8 or SF9), and allocates packets proportionally based on SF-specific Time-on-Air. Experimental results using 24 kB images reported 73.5% (SF7 and SF8) and 24.9% (SF7 and SF9) improvement in bit rate, and 42.4% (SF7 and SF8) and 19.98% (SF7 and SF9) reduction in transmission time compared to single-SF baselines. The system maintained BER below $10^{-3}$ at SNR levels above −5 dB, and is validated on a 200 m line-of-sight link with minimal hardware overhead.

### 6.2. CSMA/TDMA-Based Access Control

Wei et al. [74] implemented a time-scheduled image transmission system where three Raspberry Pi-based end devices send JPEG images (200 × 150 pixels) to a shared LoRa gateway using coordinated time slots. Each node transmitted in its assigned window, with synchronization handled via reference broadcast synchronization (RBS). Images were compressed and divided into 85 packets, sent with stop-and-wait retransmissions. Field trials over 2 km line-of-sight showed image delivery times between 54–156 s depending on SF and BW settings. The system demonstrated low-rate multi-node LoRa image transmission without inter-node interference.

Pham [75] introduced two MAC-layer mechanisms to improve LoRa image sensor deployment under ETSI 1% duty-cycle constraints. The first adapted CSMA by using LoRa’s Channel Activity Detection (CAD) feature to implement backoff and inter-frame spacing rules. The second, Long-range Activity Sharing (LAS), allowed devices to share unused airtime via gateway coordination. Both are implemented on Teensy3.2-based sensor nodes and tested in long-range deployments.

In follow-up work, [76] refined the CSMA design with CSMALoRa_new_, which replaces continuous CAD sensing with a low-power ToA-based backoff strategy. Tested on LoRa image nodes in the WAZIUP testbed (Senegal), the protocol eliminates MAC-layer collisions and reduces energy consumption under high traffic, improving robustness in dense image-sending scenarios.

### 6.3. Hybrid Network Architectures

Ta et al. [77] proposed a hybrid multimedia transmission (HMT) protocol that combines LoRa and WSN (IEEE 802.15.4 RF communication) to separate control and data paths. Scalar data and control messages were transmitted over LoRa, while JPEG2000-compressed images were relayed across a preassigned multi-hop WSN path using pipelined forwarding on multiple WSN channels. This separation eliminated WSN-level synchronization and scheduling overhead. In an 8-hop indoor testbed using STM32MP1, SX1276, and CC2630 hardware, the system achieved over 99% delivery and delivered a 24 KB image in approximately 0.3 s, reducing image latency by 25% compared to WSN-only systems.

### 6.4. Resource-Aware Chunk Allocation and Scheduling

Nurbay et al. [78] proposed a cooperative transmission scheme in which a large multimedia file was split and offloaded to neighboring end-devices based on their configuration, availability, and battery level. Each device transmitted its assigned chunks over LoRa in a time-slotted uplink, coordinated by the initiator node using a low-complexity allocation algorithm. The scheme aims to reduce total transmission time under duty-cycle and energy constraints. Experiments with ESP32-S3 and SX1276 hardware showed up to 30% time savings over equal-sized chunking, with scalability demonstrated for files up to 1024 KB and 20 cooperating nodes.

### 6.5. MAC Constraints in LoRaWAN-Based Systems

Jensen and Blaszczyk [51] experimentally evaluated the feasibility of image transmission in a LoRaWAN Class A network using JPEG2000-compressed images. Images up to 3.9 kB were fragmented according to LoRaWAN FRMPayload limits and transmitted using both confirmed and unconfirmed uplink modes across data rates DR0–DR5. The study revealed that enabling duty cycle enforcement on the end device causes transmission time to grow exponentially, with delays exceeding 6 h for 200 × 200 pixels images at DR0. Confirmed uplinks resulted in lower latency due to tighter RX1 window scheduling. These findings highlighted how LoRaWAN’s default MAC behavior imposes severe bottlenecks for multimedia data, reinforcing the need for custom or cross-layer alternatives.

### 6.6. Custom Protocol Design

Kim et al. [54] proposed a custom protocol for reliable image transmission over LoRa using segmentation, header adaptation, and stop-and-wait retransmission. The system was implemented using Raspberry Pi 4 and RFM95 modules (SX1276) with the RadioHead library, and operates on a single-hop link without a gateway. A 1.67 MB image was resized to 52 kB and segmented into 209 packets of 250 bytes each. Acknowledgment packets were exchanged per segment; retransmissions were triggered on ACK timeout. The protocol demonstrated successful recovery of all image packets in a peer-to-peer testbed, confirming its feasibility for delay-tolerant but loss-sensitive image transfers.

Fan and Ding [79] proposed a novel LoRa-based wireless visual sensor network protocol that integrates MAC-layer encryption, custom join authentication, and a layered message structure for image transmission. Their architecture supports both baseline (low-power) and continuous (low-latency) modes, with bidirectional communication between camera nodes and gateways. The protocol introduces a chaos-based symmetric encryption algorithm for both MAC and application-layer data, and includes fields for fragmentation, ACKs, and application-type tagging. While the system was not implemented or evaluated, it outlines a vertically integrated protocol stack tailored for secure visual sensing over LoRa.

## 7. Application Case Studies

**Figure 4:**
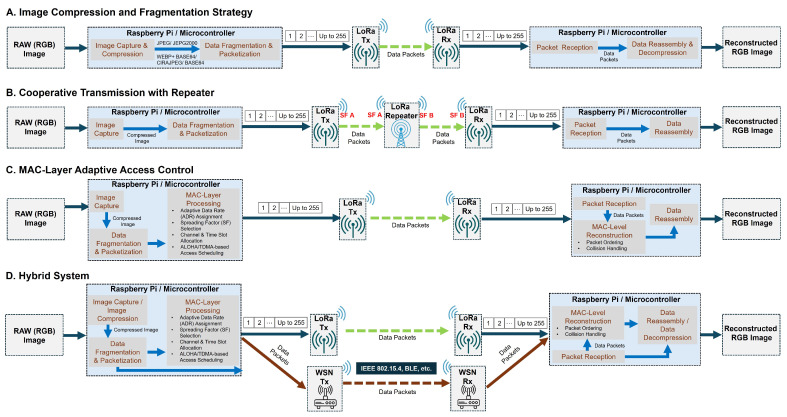
Key system-level strategies observed in real-world application case studies for LoRa-based multimedia transmission.

Real-world deployments of LoRa-based multimedia systems must contend with severe limitations in bandwidth, energy, and protocol overhead. To address these constraints, recent systems apply one or more of four core strategies discussed earlier: (1) compression and fragmentation to reduce payload size, (2) range extension through relaying, (3) MAC- or protocol-level enhancements to improve reliability or throughput, and (4) multi-module or hybrid architectures to parallelize or offload traffic (Figure 4). The following case studies are organized by domain and analyzed through this lens to highlight how practical implementations adopt these techniques in the field.

### 7.1. Smart Agriculture

Borsos [5] investigated JPEG image transmission over LoRaWAN for pest trap monitoring. A 63.8 kB image was split into 639 packets and transmitted under SF7, 868 MHz, and a 1% duty cycle. Five transmission strategies were tested, including confirmed, duplicated, and a modified MPLR [3] variant. Only the MPLR-like method achieved 100% reliability with reasonable efficiency. The system used basic compression and packetization (Strategy 1), and explored protocol-level adaptation (Strategy 3), demonstrating that LoRaWAN supports delay-tolerant, low-rate imaging.

Zaragoza-Esquerdo et al. [6] implemented a vineyard surveillance system that streams 5-s videos over LoRa at 433 MHz. Multimedia nodes were triggered by environmental anomalies and transmitted pre-encoded videos in optimized block sizes. The system used SX1278 modules with SF7, 19.2 kbps, and UART-level control. Optimal configurations yield 3.2 s transmission time for a 240p .mp4 clip with 0% packet loss. This setup reflected compression and fragmentation (Strategy 1), along with protocol-level optimization in the form of empirical block tuning (Strategy 3).

Ji et al. [7] proposed a visual monitoring system that sends only changed image patches using DSSIM-based thresholds. A 160 × 160 grayscale image was divided into 10 × 10 pixel blocks; only blocks that differ from the previous frame were transmitted. Over two hours, only 24% of cycles triggered transmissions, with an average of 14 patches per frame and SSIM > 0.955. This system heavily applied compression through selective patching (Strategy 1), but did not modify the MAC layer or used hopping or parallelism.

Tao et al. [9] proposed iCrop, which transmitted only the Top-K informative image segments from leaf images. These were compressed, packetized into 64-byte chunks, and reliably transmitted using a custom handshake protocol. The full image was avoided entirely, reducing latency to 1 min and maintaining 90% classification accuracy. This system tightly integrated compression (Strategy 1) and custom protocol design (Strategy 3), with segment prioritization based on visual salience.

Tanaka et al. [8] implemented a mushroom monitoring system that compressed images using YOLOv5-based region selection and H.265 encoding. Images were downsampled from 4.2 MB to 70 kB, then transmitted using a stop-and-wait protocol with duplicate frame detection. Indoor tests showed 6–18 min image transfer time depending on SF and BW. This work clearly used image compression and fragmentation (Strategy 1), but no higher-layer optimization was applied.

Jalajamony et al. [80,81] presented a drone-based thermal sensing system where images were segmented and processed onboard, and only metadata (dry patch information) was transmitted via LoRa. Transmission was point-to-point using SX1276 at 915 MHz. The drone transmitted georeferenced alerts within 5 s of detection. While no images were transmitted, the work applied a compression-equivalent strategy (Strategy 1) by pre-processing and transmitting only essential data.

Cai et al. [82] implemented a hybrid AIoT system for vertical farming that integrated BLE-based sensors with LoRaWAN-enabled camera nodes. Daily 640 × 480 images are transmitted via LoRaWAN (SF7, 250 kHz, Class A) to a backend server for AI-based plant analysis. While the paper confirmed image delivery via LoRaWAN, it omits details on segmentation, compression, or packet structure. The architecture applied basic image capture and transmission (Strategy 1) and leveraged a hybrid BLE–LoRaWAN design for combining short- and long-range communication (Strategy 4), making it a practical framework for low-rate, image-driven crop monitoring.

Brazhenenko et al. [4] proposed a fog-assisted smart agriculture framework intended to support scalable, low-latency farm monitoring using LoRa as the wireless backbone. The system architecture was evaluated via iFogSim, comparing traditional cloud-based models with a hierarchical fog-layer approach. While LoRa was referenced (433 and 868 MHz, 125–500 kHz), no actual LoRa hardware or multimedia data transmission was simulated. The work remained conceptual and did not engage any of the core multimedia-enabling strategies identified in this survey.

Villarreal et al. [83] simulated LoRa signal propagation across rural Colombian terrain using Xirio radio planning software to assess feasibility for image transmission in agriculture. The study provided signal strength estimates and optimal placement strategies over distances up to 8 km but did not simulate image fragmentation, transmission, or reconstruction. The work remained conceptual and did not engage any of the core multimedia-enabling strategies identified in this survey.

Angin et al. [84] proposed AgriLoRa, a simulation-based smart agriculture framework combining LoRaWAN sensor data and cloud-based image analysis within a digital twin architecture. The LoRaWAN WSN transmitted only scalar environmental data in Class A mode, simulated using the FLoRa framework. Aerial imagery was assumed to be available directly in the cloud and analyzed using a MobileNet-based CNN trained on PlantVillage. While the system evaluated LoRa parameters like spreading factor and power, no image data was transmitted or simulated over LoRa. As such, the work did not apply any of the four core strategies for multimedia transmission.

### 7.2. Projectile Tracking

Kim et al. [85] developed a LoRa-based projectile tracking system that avoids transmitting full images by sending only compressed coordinate metadata of bullet impacts. An 11 kB reference image is initially transmitted using the MPLR protocol, reducing transmission time from 11 min to 80 s over a 300 m LoS link. Once the image is received, real-time impact locations are computed onboard and transmitted as a few bytes. This system applied aggressive data minimization and metadata extraction (Strategy 1), paired with a custom reliable multi-packet protocol (Strategy 3), to achieve robust and timely visual feedback in bandwidth-constrained outdoor shooting environments.

### 7.3. Environmental Monitoring

García et al. [10] deployed a heterogeneous LoRa-based wireless multimedia sensor network (WMSN) for long-term environmental monitoring in Doñana National Park. Each node integrated a low-power STM32 microcontroller with a Raspberry Pi 3 to manage energy and handle multimedia data, including images from a visible-light camera and a FLIR thermal sensor. Images were preprocessed locally before LoRa transmission. This design employed edge-side compression and data reduction (Strategy 1) and microcontroller-processor task scheduling, achieving autonomous operation for over a year on solar power without physical maintenance.

Zurek et al. [13] developed a LoRa-enabled pest detection system that used an OpenMV board to capture and analyze images of sticky traps. A lightweight onboard DL model cropped relevant image segments before transmission, reducing data volume. Transmission of 4 kB files over LoRa was completed in 6 s under real orchard conditions, with final integrity verified using SHA-based hash checks. The system adopted onboard data pruning (Strategy 1) and reliability mechanisms (Strategy 3) to support low-power pest monitoring in constrained agricultural settings.

Trinchero et al. [14] proposed a dual-radio WMSN node combining LoRaWAN and GPRS to monitor plant growth over an entire season. A low-power STM32L0 handled telemetry via LoRaWAN, while an ESP32-S3 managed high-resolution image capture and upload over GPRS. LoRaWAN was used to coordinate imaging schedules, enabling the GPRS module to remain idle most of the time. While images were not transmitted over LoRa, the system applied control-plane optimization (Strategy 3) and a hybrid radio design (Strategy 4) to minimize energy use, achieving 300 days of operation on two AA batteries.

Almstedt et al. [15] deployed a LoRa-enabled pest monitoring system combining stationary and sticky trap cameras with edge ML and blockchain-backed logging. OpenMV boards cropped image regions pre-inference, and LoRa is used to transmit 4 kB cropped packets across orchards, with reliability preserved even under foliage. A future mesh LoRa design with synchronization and negotiation phases is proposed. This system demonstrated efficient image cropping (Strategy 1), challenging terrain testing, protocol coordination proposals.

Zinonos et al. [16] investigated LoRa-based transmission of grape leaf images for disease detection. Images were downscaled and converted to grayscale, then fragmented into 85-byte LoRa packets. A simulation tool injected packet loss patterns into image segments, and CNNs (ResNet50, MobileNetV2) were trained to tolerate missing data. Grad-CAM visualizations confirmed that relevant features remained detectable even under 50% corruption. This work explored compression via preprocessing (Strategy 1) and robustness to partial packet loss (Strategy 3), validating ML-ready LoRa transmission in agricultural disease monitoring.

### 7.4. Resilient Monitoring Systems

IFT [66] has been validated through outdoor deployments at Norfolk State University, showing improved PRR and SNR in semi-obstructed environments. The study demonstrates that dual-receiver LoRa systems can deliver large images more reliably, making them suitable for visual IoT applications such as environmental monitoring.

Cai et al. [11] deployed an AIoT-based LoRaWAN camera system for disaster-resilient image monitoring. ESP32 camera nodes with IP67 ratings transmitted daily compressed images via LoRaWAN, using the CIRA framework to apply full compression, discrepancy-based filtering, and region-of-interest selection. Advanced retransmission and history-based reconstruction ensured full image recovery under packet loss. Tested for 61 days in Hong Kong (including two typhoons), the system achieved 100% image integrity using hybrid recovery. This deployment leveraged advanced compression and recovery (Strategy 1), and robust protocol logic for retransmission (Strategy 3), validating LoRa’s use in smart city resilience applications.

Marrara et al. [12] deployed a multi-hop LoRa network for flood-risk infrastructure monitoring in Italy’s Sant Agata basin. Four SX1262 based nodes relayed 640 × 480 images (∼20 kB base64-encoded) and rainfall metadata over 1 km using a hop-to-hop JSON payload structure. The mesh network maintained communication despite node outages and improved signal quality over single-hop baselines. This architecture applied basic image fragmentation (Strategy 1) and range extension via multi-hop relay (Strategy 2), enabling continuous infrastructure surveillance in low-connectivity regions.

### 7.5. Surveillance Systems

Fort et al. [1] developed a motion-triggered video surveillance unit (VSU) that transmitted compressed images over LoRaWAN. The system used Raspberry Pi 4 for processing and Nucleo-L073RZ for LoRaWAN control, transmitting WebP-compressed and base64-encoded frames (∼3.5–4 kB) split into 15–18 packets. Under the 1% duty cycle, transmission time per frame was 9–11 min. The system implemented compression and fragmentation (Strategy 1) but relied on standard LoRaWAN behavior for scheduling and reliability. Despite high latency, the architecture supported periodic frame delivery for remote surveillance in bandwidth-constrained environments.

Rodrigues et al. [2] implemented a facial recognition surveillance system using LoRa. Motion-triggered ArduCAM modules captured JPEG images (up to 1600 × 1200), which were fragmented into 256-byte packets and transmitted over 433 MHz LoRa. A dedicated router mediated traffic between sensor nodes and a recognition server, which used Dlib and a ResNet-based model. The design applied compression and fragmentation (Strategy 1) and introduced a MAC-level relay architecture for multi-node coordination (Strategy 3), enabling scalable LoRa-based facial recognition for edge surveillance scenarios.

Pham [86] presented a low-cost LoRa image sensor for long-range visual surveillance. A uCamII module captured 128 × 128 grayscale frames, which were compressed using a lightweight JPEG-like encoder with block interleaving to improve packet loss resilience. Compressed images (∼911 bytes at QF = 10) were split into 240-byte LoRa packets and transmitted using an SX1276-based inAir9 module. Even under 20–40% simulated packet loss, reconstructed images retained acceptable visual quality (PSNR ≈ 25.3 dB). The system consumed just 1.254 J per capture–transmit cycle and supported hourly operation for up to 268 days on 4 × AA batteries. This work applied low-complexity compression and robust fragmentation (Strategy 1) for energy-efficient, packet-tolerant LoRa visual monitoring.

### 7.6. Comparative Summary

Across agriculture, surveillance, environmental sensing, and infrastructure monitoring, LoRa has been deployed in diverse image-enabled systems.

Table 3 summarizes the systems reviewed in this section by domain and the strategies they implement. Most systems apply some form of compression or fragmentation, often with selective transmission of patches, metadata, or compressed blocks. Protocol-level enhancements and multi-hop topologies appear in more recent works targeting reliability and scalability. Few deployments implement concurrent LoRa streams or dynamic cross-layer optimization, reflecting practical complexity and energy constraints in current LoRa hardware.

## 8. Open Research Problems

This section identifies key unresolved issues and underexplored directions emerging from current research on LoRa-based multimedia transmission. Each subsection highlights a specific research gap supported by examples from recent deployments or simulations.

### 8.1. Spectrum-Aware Transmission Using Frequency Diversity

Despite strict regional regulations on sub-GHz ISM bands-such as the 1% duty cycle under ETSI in Europe or the 400 ms dwell-time limit imposed by the FCC in the United States-most LoRa-based image transmission systems do not explicitly consider these constraints. This oversight limits their scalability and real-world deployability, especially for multimedia applications requiring large or frequent data transfers.

One underexplored approach is to exploit frequency diversity within the allowed ISM bands. By fragmenting images and transmitting chunks across different LoRa frequencies (e.g., 868.1, 868.3, 868.5 MHz), systems can comply with per-channel duty cycle restrictions while effectively increasing the aggregate throughput. This strategy resembles application-layer frequency-hopping spread spectrum (FHSS), but is specifically adapted to fragmented multimedia payloads.

Some recent systems hint at this design space. For example, Wei et al. [72] demonstrated a segmented transmission method using different spreading factors across relay nodes. Similarly, the Interleaved Frequency Transmission (IFT) approach [66] employs a dual-receiver architecture tuned to adjacent frequencies (915 and 914.5 MHz), enabling pseudo-parallel reception without altering the LoRa PHY. Field experiments showed that IFT can reduce end-to-end image transmission time by 50% while lowering transmitter energy consumption by up to 59%. These results suggest that spectrum-aware, multi-frequency or multi-receiver strategies represent a promising but underexplored avenue for future multimedia IoT deployments.

**Open Problem:** How can LoRa image transmission protocols be designed to dynamically fragment and distribute payloads across multiple ISM sub-bands, enabling concurrent or pipelined delivery while maintaining compliance with regional regulatory limits?

### 8.2. Toward a Standardized Multimedia Protocol Stack for LoRa

Most systems bypass or heavily modify the LoRaWAN stack to meet the demands of image or video transmission. Solutions like Multi-Packet LoRa (MPLR) [3], CIRAJpeg [59], or custom handshaking protocols [9] demonstrate fragmented efforts. A unified, lightweight, multimedia-aware stack that supports fragmentation, loss tolerance, and optional acknowledgments under regulatory constraints remains absent.

**Open Problem:** What architectural and protocol-layer abstractions are needed to standardize multimedia transmission over LoRa while maintaining flexibility for diverse sensing and regulatory contexts?

### 8.3. Scalable Cross-Layer Frameworks for LoRa Multimedia Networks

Pham’s integrated architecture for image transmission over LoRa [75,76,86] remains one of the most technically complete examples of cross-layer design. It couples a custom JPEG-like encoder with interleaved block ordering, LoRa-aligned fragmentation, and a CSMA-based MAC protocol that utilizes Channel Activity Detection (CAD) or slot-based backoff depending on traffic conditions. These techniques are deployed in real-world field tests under duty-cycle constraints and packet loss scenarios, achieving robust performance and energy efficiency.

Despite this depth, Pham’s approach has not been generalized. Most recent systems optimize only one layer at a time and rarely consider MAC-aware compression or energy-driven scheduling. Moreover, no work has demonstrated cross-layer techniques at scale across multi-hop or relay-assisted networks.

**Open Problem:** Can the principles of cross-layer compression and MAC coordination be extended to scalable, multi-node LoRa multimedia networks that operate under realistic field constraints and heterogeneous node capabilities?

### 8.4. Scalability in Multi-Node Image-Sending LoRa Networks

Most real-world deployments are single-node or point-to-point setups. Exceptions like MPLR with reservation [3] or LoRa-CSMA [76] hint at the complexity of scaling LoRa for dense image sensor networks. Research into contention resolution, image-aware scheduling, and gateway fairness is essential for LoRa to support high-density, real-time multimedia sensing applications.

**Open Problem:** How can LoRa-based multimedia systems be scaled to support dozens or hundreds of image-transmitting nodes while minimizing contention, ensuring fairness, and maintaining regulatory compliance?

### 8.5. Loss-Resilient Encoding Without Retransmission

LoRaWAN Class A does not support fast acknowledgments or in-order retransmission. Some systems tolerate loss by design; e.g., the DSSIM patch-based model [7] or packet-loss simulations in [16], but few encode redundancy explicitly. There is a need for progressive or error-tolerant image encoding schemes suited to lossy LoRa channels, with minimal decoding complexity at the gateway.

**Open Problem:** What are the most effective lightweight encoding schemes for LoRa that can gracefully degrade under loss, minimize decoding overhead, and operate without feedback or retransmission support?

### 8.6. Energy-Aware Runtime Adaptation and Profiling

While some systems provide energy estimates (e.g., 1.254 J/image in [86], 300 mW transmission in [81]), few adapt dynamically to energy state. Real-time tuning of resolution, compression level, or transmission interval based on remaining power or forecasted load is largely unexplored. Integrating energy-awareness across layers remains an open design space.

**Open Problem:** How can LoRa multimedia nodes adapt image capture, compression, and transmission in real-time to maximize operational lifetime under varying battery and environmental conditions?

### 8.7. Compression Techniques Tuned for Machine-Readable Transmission

Many works optimize for PSNR or SSIM, despite final image use being for classification or detection. For example, Sachinda et al. [63] prioritize ML inference accuracy even with degraded image quality, while [16] show CNN robustness under corrupted packets. Yet, compression schemes explicitly tuned for downstream ML tasks over LoRa remain rare. Designing encoders that optimize task-specific performance under bandwidth constraints is a promising direction. Brazhenenko et al. [87] argue that conventional PSNR-based metrics are insufficient and propose defining image integrity through interpretability, whether a downstream ML model produces consistent output from compressed or degraded input.

**Open Problem:** How can image encoders be designed or adapted to prioritize task-level ML inference performance over traditional metrics like PSNR, especially in lossy LoRa environments?

## 9. Conclusions

This paper presented a comprehensive survey of image and video transmission over LoRa networks, covering compression methods, cooperative transmission strategies, and MAC/protocol-level optimizations. A structured taxonomy was proposed and applied to over 40 systems, including real-world deployments in agriculture, surveillance, and environmental monitoring.

While progress has been made across individual layers, no unified solution addresses the trade-offs between image quality, energy efficiency, and regulatory compliance. Unresolved problems remain in cross-layer coordination, ML-aware compression, and adaptive scheduling. This survey aims to serve as a reference for future research on scalable and reliable multimedia systems over LoRa.

## Figures and Tables

**Table 1:** Summary of Key Constraints for Multimedia over LoRa.

Constraint Type	Source	Description
Max Payload Size	LoRa PHY	Up to 255 bytes per packet; requires fragmentation for even small images
ToA	LoRa PHY (SF, BW, CR)	Increases exponentially with SF; limits available airtime under regulatory rules
Bitrate	PHY Limits	Typically under 6.5 kbps; practical upper bound of 35 kbps with SF6 and 500 kHz
Duty Cycle/Dwell Time	FCC/ETSI	1% duty cycle (EU) or 400 ms dwell time (US) restricts transmission frequency
Fragmentation Overhead	LoRa PHY	Requires division of images into 20–100 small packets due to LoRa’s 255-byte limit, increasing header overhead, energy usage, and risk of packet loss
Link Reliability	LoRa PHY	No native retransmission or in-order delivery; complicates reassembly
Latency Tolerance	Application Layer	Some use cases (e.g., surveillance) require near-real-time response
Energy Constraints	Edge Devices	Long ToA and frequent retries deplete battery life in mobile nodes

**Table 2:** Comparison of Compression Techniques for LoRa-Based Image Transmission.

Paper	Compression Type	Key Results	Evaluation	Notes
Kirichek et al. [55]	Conventional JPEG, JPEG2000	JPEG2000 outperforms JPEG in PSNR and packet loss	Hardware Tested	Grayscale image; includes subjective quality rating
Correia et al. [57]	Conventional JPEG	2.6–8.4 KB JPEG images; 24–26 min transmission using 25–50 B packets	Hardware Tested	ESP32-CAM + MKR WAN 1310; TTN; stop-and-wait ARQ; 2.5 km LoRaWAN range
Wei et al. [50,58]	Conventional WebP+Base64, JPEG, H.264	WebP+Base64 transmits faster with acceptable quality	Hardware Tested	Low-res image (200 × 150); Base64 introduces overhead
Obeng et al. [52]	Conventional JPEG, Base64	JPEG is faster; Base64 more robust	Hardware Tested	Image sizes up to 164 KB; lacks per-image PSNR reporting
Zhang et al. [59]	Conventional Headerless JPEG (CIRA)	CIRA improves recovery under packet loss	Simulation	Assumes LoRaWAN; uses metadata and preview frame
Guerra et al. [60]	Transform Wavelet + Huffman	Slightly better compression than JPEG2000	Simulation	Tested on RPi; minor compression gain; no packetization
Haron et al. [61]	Transform DCT	49× compression; PSNR ≈ 25.7 dB	Simulation	MATLAB-only; grayscale snail images; no real hardware
Chaparro et al. [62]	Compressive Sensing Wavelet + CS + TwIST	128 × 128 pixels image in 4 packets; PSNR up to 30 dB	Simulation	SDR-based; high compute complexity; sensitive to loss
Sachinda et al. [63]	ML-Based JPEG + Classifier	<10 KB images maintain ≥90% ML accuracy	Hardware Tested	LoRa tested at 433 MHz; quality tuned for ML not humans
Körber et al. [64]	ML-Based GAN-based compression	0.036 bpp; 99% lower memory; BPG-like quality	Simulation	No LoRa hardware; focus on encoder efficiency
Shiddiq et al. [65]	ML-Based CNN Autoencoder	PSNR up to 30.5 dB; 728 B latent vector	Simulation	80 × 80 pixels input; bandwidth constraints simulated only

**Table 3:** Summary of Real-World LoRa Deployments with Multimedia Capabilities.

Paper	Application Domain	Strategies Applied	LoRa Configuration	Notes
Borsos [5]	Agriculture	1, 3	LoRaWAN, SF7, 868 MHz	JPEG split into 639 packets; image/day feasible
Chen et al. [3]	Agriculture	1, 3	SF7–SF11, BW125–500 kHz	MPLR + reservation improves fairness in 20-node tests
Tao et al. [9]	Agriculture	1, 3	SF7, 250 kHz	Top-K segment selection + custom handshake protocol
Ji et al. [7]	Agriculture	1	Custom LoRa; no SF given	DSSIM-based patch filtering; 24% cycles transmit
Zaragoza-Esquerdo et al. [6]	Agriculture	1, 3	433 MHz, UART LoRa	Event-driven video block transmission
Tanaka et al. [8]	Agriculture	1	SF7–SF9, H.265	YOLOv5-based region filtering + H.265 encoding
Jalajamony et al. [80,81]	Agriculture	1, 2	SX1276, SF7–SF12, 915 MHz	Drone transmits thermal metadata; tested to 778 m, 300 mW power profile
Cai et al. [82]	Agriculture	1, 4	LoRaWAN, SF7	BLE + LoRaWAN; daily image + sensor fusion
Zurek et al. [13]	Env. Monitoring	1, 3	SX1276, ≤200 m	Onboard DL selects trap crop; 4 kB in 6 s
García et al. [10]	Env. Monitoring	1	868 MHz; hybrid MCU+Pi	Solar WMSN, thermal + visual sensors
Trinchero et al. [14]	Env. Monitoring	3, 4	LoRaWAN + GPRS	STM32+ESP32 split architecture; seasonal ops
Almstedt et al. [15]	Env. Monitoring	1	SX1276, mesh (planned)	Multi-sensor BMSB mesh with edge ML + blockchain
Zinonos et al. [16]	Env. Monitoring	1, 3	SF7–SF12 (simulated)	CNN classification resilient to packet loss
Marrara et al. [12]	Resilient Monitoring	1, 2	SX1262, 1 km multi-hop	Check-dam image monitoring + failure-tolerant mesh
Cai et al. [11]	Resilient Monitoring	1, 3	LoRaWAN, TTN, SF7	CIRA compression + retransmission; 100% recovery
Kim et al. [85]	Projectile Tracking	1, 3	915 MHz, P-MPLR	Reference image + impact metadata; sub-5 s latency
Fort et al. [1]	Surveillance	1	LoRaWAN, base64 frames	WebP image upload; 9–11 min per event
Rodrigues et al. [2]	Surveillance	1, 3	433 MHz, 256 B packets	Router-managed JPEG fragments for face recognition
Pham [86]	Surveillance	1	SF12, 240 B packets	JPEG-like with interleaving; 268-day runtime node

## Data Availability

Data are contained within the article.

## Abbreviations

The following abbreviations are used in this manuscript:

bps	bits per second
CR	Coding Rate
CSMA	Carrier Sense Multiple Access
CSS	Chirp Spread Spectrum
DCT	Discrete Cosine Transform
FCC	Federal Communications Commission
GAN	Generative Adversarial Networks
IFT	Interleaved Frequency Transmission
Kbps	Kilobits per second
LoRaWAN	Long Range Wide Area Networks
LPWAN	Low Power Wide Area Networks
MAC	Media Access Control
MPLR	Multi-Packet LoRa
MSE	Mean Squared Error
PHY	Physical
PRR	Packet Recovery Rate
PSNR	Peak Signal to Noise Ratio
RSSI	Received Signal Strength Indication
SDR	Software Defined Radio
SSIM	Structural Similarity Index
ToA	Time on Air
TTN	The Things Network

## Appendix A

### Appendix A.1. ToA Derivation

A step-by-step derivation of the Time-on-Air (ToA) for the adopted LoRa packet configuration is presented. The parameters used are listed in Table A1. The equations for computing ToA are adopted from the official Semtech SX1276 datasheet [17].

**Table A1:** LoRa Packet Configuration Parameters.

Parameter	Value	Units
Spreading Factor (SF)	6	dimensionless
Bandwidth (BW)	500	kHz
Payload Size (PL)	240	bytes
Coding Rate (CR)	1 (⇒ 4/5)	–
Cyclic Redundancy Check (CRC)	1 (⇒ Enabled)	–
Low Data Rate Optimization (DE)	0 (⇒ Disabled)	–
Preamble Length ($n_{\mathrm{preamble}}$)	8	symbols

1. Symbol duration ($T_{\mathrm{symbol}}$):
  (A1) $$T_{\mathrm{symbol}}=\frac{2^{SF}}{BW}=\frac{2^{6}}{500000}=0.128\;\mathrm{ms}.$$
2. Preamble duration ($T_{\mathrm{preamble}}$):
  (A2) $$\begin{array}{cc} T_{\mathrm{preamble}} & =\left(n_{\mathrm{preamble}}+4.25\right)\times T_{\mathrm{symbol}}\\ \; & =(8+4.25)\times 0.128=1.568\;\mathrm{ms}. \end{array}$$
3. Payload symbols ($n_{\mathrm{payload}}$):
  (A3) $$n_{\mathrm{payload}}=8+\max\left(\left\lceil \frac{8PL-4SF+28+16CRC-20IH}{4(SF-2DE)}\right\rceil \left(CR+4\right),\;0\right)$$
  With PL = 240, SF = 7, H = 0, DE = 0, CR = 1:
  (A4) $$\begin{array}{cc} n_{\mathrm{payload}} & =8+\left\lceil \frac{8\times 240-4\times 6+28+16-0}{4\times (6-0)}\right\rceil \times 5\\ \; & =8+\left\lceil \frac{1940}{24}\right\rceil \times 5=8+81\times 5=413. \end{array}$$
4. Payload duration ($T_{\mathrm{payload}}$):
  (A5) $$\begin{array}{cc} T_{\mathrm{payload}} & =n_{\mathrm{payload}}\times T_{\mathrm{symbol}}\\ \; & =413\times 0.128=52.864\;\mathrm{ms}. \end{array}$$
5. Total packet time-on-air ($T_{\mathrm{packet}}$):
  (A6) $$\begin{array}{cc} T_{\mathrm{packet}} & =T_{\mathrm{preamble}}+T_{\mathrm{payload}}\\ \; & =1.568+52.864=54.432\;\mathrm{ms}. \end{array}$$
6. Transmission Bit Rate ($Tx_{\mathrm{Kbps}}$):
  (A7) $$\begin{array}{ccc} Tx_{\mathrm{Kbps}} & =\frac{PL}{T_{\mathrm{packet}}}\; & =\frac{240}{54.432}=35.27\;\mathrm{ms}. \end{array}$$

### Appendix A.2. Comparative Summary of LoRa-Based Multimedia Sensing Platforms

**Table A2:** Summary of hardware, transmission performance, and deployment characteristics in LoRa-based multimedia sensing platforms. (Part 1).

Ref.	Authors	Sensor Used	Processor Platform (TX/RX)	Tx Time	Energy Consumed	Power Source	LoRa IC	LoRa Freq.	Region
[55]	Kirichek et al.	Camera (160 × 120), Microphone	Raspberry Pi Zero + STM32L	Not specified	Not reported	DC via Pi + UART	SX1267	868 MHz	Lab (Russia)
[50]	Wei et al.	Camera (200 × 150 px)	Raspberry Pi 3 B+	25.7–47.7 s/image	TP = 17 dBm (current not specified)	DC via Pi GPIO	SX1276	868 MHz	Outdoor (1.5 km), Taiwan
[56]	Jebril et al.	Adafruit TTL JPEG Camera	Arduino MEGA/Uno	1–14 min (SF7–SF12)	TP = 14 dBm (not specified)	12 V battery + solar	RN2903	921.9 MHz	Outdoor (Malaysia)
[57]	Correia et al.	ESP32-CAM (OV2640)	ESP32-CAM + MKR WAN 1310	24–26 min/image	Not reported	USB power	MKR WAN 1310	868 MHz (assumed)	Urban (Portugal)
[36]	Zhang et al.	Simulated 100 KB image block	STM32 + Zynq + SX1278	47.5 s @ 21, 875 bps	TP = 20 dBm	12 V battery + FRP antenna	SX1278	433 MHz	Outdoor (Urban + Mountain, China)
[51]	Jensen and Blaszczyk	Raspberry Pi Camera (JPEG2000)	Raspberry Pi 0 + ESP32 + SX1276	34 s; DR5	Not reported	USB + DC supply	SX1276	868 MHz	Lab (Denmark)
[52]	Obeng et al.	JPG/PNG (3–164 KB)	Raspberry Pi 4 + SX1276 (RFM95W)	3–20 s (JPEG); longer for Base64	Not reported	5V via Pi GPIO	SX1276	868 MHz	Indoor short-range test (USA)
[65]	Shiddiq et al.	RGB camera (80 × 80 px)	Raspberry Pi 4 + CNN autoencoder (Keras)	0.16–0.35 s/image (simulated)	Avg. PSNR 24.6 dB, SSIM > 0.9	DC + Pi GPIO	SX1276 (sim.)	915 MHz	Simulated + lab (Indonesia)
[64]	Körber et al.	RGB (480 × 640)	MobileNetV2 (TinyML) + Cloud GAN	∼0.16–0.35 s (simulated)	0.072 bpp, FID: 39.5	1 MB SRAM (QAT)	N/A (LPWAN-focused)	Simulated LPWAN	Indoor (Germany)
[70]	Gao et al.	Camera (not specified)	STM32 (Cortex M0) + Zynq 7000	∼99 ms/packet @ DR = 21,875 bps	Not reported	Battery (TX), DC 220 V–12 V (RX)	SX1278	433 MHz	Outdoor (Beijing, China)
[74]	Wei et al.	Camera (200 × 150 px)	Raspberry Pi 3B (3 TX) + RPi 3B (Gateway)	54–156 s/image	Not reported	DC via USB	SX1276	868 MHz	Outdoor (2 km), Taiwan
[12]	Marrara et al.	Camera (640 × 480), Rain Sensor	Heltec WiFi LoRa 32 v3 (SX1262)	Not Specified	Not Specified	Not Specified	SX1262	Not Specified	Italy (Reggio Calabria)
[1]	Fort et al.	Camera Module v2.1 (Raspberry Pi)	Raspberry Pi 4 + STM32L073 + RAK831 (Gateway)	9-11 min/image (15–18 packets)	Not Specified	Mains Power	RFM95 (HopeRF)	Not Specified	Italy (Indoor Lab)

**Table A3:** Summary of hardware, transmission performance, and deployment characteristics in LoRa-based multimedia sensing platforms. (Part 2).

Ref.	Authors	Sensor Used	Processor Platform (TX/RX)	Tx Time	Energy Consumed	Power Source	LoRa IC	LoRa Freq.	Region
[59]	Zhang et al.	OV2640 (QVGA 320 × 240)	ESP32-CAM + CubeCell AB02 (SX1262) + CIRA Server	∼14–16 packets/image	Not reported	3.7V 300mAh Li-ion + power-gated ESP32	SX1262	923 MHz	Simulated Hong Kong (LoRaWAN)
[72]	Wei et al.	JPEG (200 × 150 px)	Raspberry Pi 3B + SX1276	48 s (1-to-1), 26 s (3-to-3)	Not reported	5V mobile power	SX1276	868 MHz	Outdoor (1.5 km), Taiwan
[63]	Sachinda et al.	ESP32-CAM (OV2640)	ESP32-CAM + RA-02 (SX1278) + Edge Laptop	<20 s (JPEG quality ≤10%)	TP = 14 dBm, SF6, BW = 500 kHz	3.7 V Li-ion + DC supply	SX1278	433 MHz	Indoor testbed (Sri Lanka)
[75]	Pham	uCamII/III (QVGA JPEG)	Teensy 3.2 MCU + SX1276	4–5 packets/image (Q = 10); ToA ≈ 9.1 s (SF12, 125 kHz)	Not reported	4xAA batteries (1+ year at 1 img/hr)	SX1276	868 MHz	Field test (France)
[58]	Wei et al.	Camera (200 × 150 px)	Raspberry Pi 3B + SX1276	25.7 s (WebP), 47.7 s (JPEG), 32.9–39.2 s (H.264)	Not reported	DC via USB	SX1276	868 MHz	Outdoor (1.5 km), Taiwan
[86]	Pham	uCamII (128 × 128, grayscale)	Teensy 3.2 (Cortex-M4) + SX1276 (inAir9)	∼4–23 pkts/img (Q = 10–90)	∼1.254J (encode + packetize @96MHz)	4xAA battery (268 days @1 img/hr)	SX1276	868 MHz	Urban field test (France)
[54]	Kim et al.	Resized Image (1.67 MB → 52 kB)	Raspberry Pi 4 + RFM95 (SX1276)	209 packets (w/retransmissions)	Not reported	5V DC	SX1276 (RFM95)	915 MHz	Lab (USA)
[69]	Kim et al.	JPG (414–419 bytes)	Raspberry Pi 4 + RFM95 (SX1276)	2 packets/image	Not reported	5V via Pi USB	SX1276	915 MHz	Indoor Lab (USA)
[76]	Pham	uCamII (128 × 128 grayscale)	Teensy32 + Arduino Pro Mini + SX1276	4–5 packets/image (900–1200 B total)	Duty-cycle compliant; CSMA energy saving: 2x	4x AA batteries	SX1276	868 MHz	Outdoor (Senegal, WAZIUP deployment)
[2]	Rodrigues et al.	ArduCAM Mini 2MP + PIR Sensor	Arduino UNO + E32-433T20DC + PC (Python)	Not Specified	Not Specified	Battery	SX1278	433 MHz	Brazil (João Pessoa, Indoor Test)

**Table A4:** Summary of hardware, transmission performance, and deployment characteristics in LoRa-based multimedia sensing platforms. (Part 3).

Ref.	Authors	Sensor Used	Processor Platform (TX/RX)	Tx Time	Energy Consumed	Power Source	LoRa IC	LoRa Freq.	Region
[71]	Torres et al.	OV7670 (640 × 480)	Arduino MEGA + UNO + E220-900T22D	9–25 s/image (1 km)	Full cycle ≈ 97200 mJ	9 V 3000 mAh battery	E220-900T22D	900 MHz	Outdoor (Lima, Peru)
[77]	Park et al.	OV2640 camera + scalar sensors	ATmega128RFA1 dual-radio node + Master node	Not specified (LoRa TDMA, 4–5 hops)	Not reported	DC + batteries	SX1276	915 MHz	University building (Korea)
[78]	Nurbay et al.	Image chunks (10–1024 KB total)	ESP32-S3 (6 EDs) + RPi Gateway	1–3.5 min (10 KB test)	Not explicitly reported; optimization-based	3.7V Li-ion (ESP32-S3)	SX1276	868 MHz	Indoor testbed (Kazakhstan)
[4]	Brazhenenko et al.	Not Specified	Fog Node with STM32 (LoRa Gateway)	Not Specified	Not Specified	Solar	Not Specified	868/433 MHz	Morocco (assumed based on affiliation)
[5]	Borsos	JPEG Image (60KB)	Not Specified	∼1.5 h/image	Not Specified	Not Specified	Not Specified	868 MHz	Hungary (assumed based on affiliation)
[6]	Zaragoza-Esquerdo et al.	Grayscale Video	Atmega2560 (TX)/ESP32 (RX)	∼5–25 s/frame	<200 mW during TX	9V@1A DC-DC converter	SX1278	433 MHz	Rural (Spain, assumed based on affiliation)
[7]	Ji et al.	Pi Camera (16 × 160 px)	Raspberry Pi 3B/Arduino Uno	Not Specified	∼5.22 s/update	Not Specified	Battery/Not specified	LoRa Shield (unspecified)	Field (Japan)
[3]	Chen et al.	Brinno TLC200 Pro (Images)	Raspberry Pi + Dragino LoRa Hat	∼5–51 s/image (MPLR)	Not Specified	Battery	SX1276	902–928 MHz	Canada
[80]	Jalajamony et al.	Thermal + RGB Camera	Raspberry Pi 4	∼<1 min/metadata	∼300 mW TX, 60 mW RX	Battery	SX1276	915 MHz	USA
[83]	Villarreal et at.	Not implemented (simulation)	Simulation only	Not simulated	Not Applicable	Not Applicable	Not Applicable	Simulation	Colombia (simulated)
[84]	Angin et al.	Drone RGB imagery (cloud input)	Simulation only	Not simulated	Simulated only	Not Applicable	SX1272/73 (simulated)	868 MHz	Simulated
[81]	Jalajamony et al.	Thermal Camera	Raspberry Pi 4	∼<1 min/metadata	∼300 mW TX, 60 mW RX	Battery	SX1276	915 MHz	USA

**Table A5:** Summary of hardware, transmission performance, and deployment characteristics in LoRa-based multimedia sensing platforms. (Part 4).

Ref.	Authors	Sensor Used	Processor Platform (TX/RX)	Tx Time	Energy Consumed	Power Source	LoRa IC	LoRa Freq.	Region
[8]	Tanaka et al.	Raspberry Pi Camera + Sensors	Raspberry Pi 3B	∼6–18 min/image	Solar-powered, not quantified	Solar battery	Not Specified	433 MHz	Japan (Satoyama)
[9]	Tao et al.	RGB Camera (Segmented)	Raspberry Pi 3B + Heltec WiFi LoRa 32	∼1 min (Top-K segments)	∼3.6% packet loss, no energy data	Battery (assumed)	Heltec WiFi LoRa 32 V2	915 MHz	Agricultural field
[82]	Cai et at.	CIRA Camera (640 × 480)	STM32F429 (TX)	Not Specified	Not Specified	Not Specified	RAK811	868 MHz	Indoor farm (China, assumed based on affiliation)
[85]	Kim et al.	HQ Camera, Microphone	Raspberry Pi 4B (8 GB RAM, both nodes)	∼80 s (1 image), <5 s (coordinates)	∼300 mW TX	Battery	SX1272	915 MHz	Urban & rural (USA)
[10]	Garcia et al.	8MP CMOS + Thermal Camera	STM32L162RD + Raspberry Pi 3	Not Specified	Not Specified	Battery + Solar	SX1276	868 MHz	Spain
[13]	Zurek et al.	OpenMV Camera	OpenMV Board	6 s for 4 KB image	Not Specified	Battery	SX1276	869.7 MHz	Italy (Orchard)
[14]	Trinchero et al.	ArduCAM Mega SPI (5MP JPEG)	STM32L0 (Murata 1SJ) + ESP32-S3	25 s for 200 KB image (GPRS)	8.31 mAh/day (LoRa + GPRS)	2× AA Battery	SX1262	Not Specified	Italy (Verrua Savoia)
[15]	Almstedt et al.	Sticky Trap Camera + Raspberry Pi HQ Camera	OpenMV + Raspberry Pi 4 + Intel NUC	Not Specified	Not Specified	Sticky Trap: Solar, Rest: Public Power Supply	SX1276 (mentioned for evaluation)	Not Specified	Italy (Carpi, Modena)
[16]	Zinonos et al.	Grayscale Camera (255x255 pixels)	Arduino UNO + Dragino LoRa Shield	765 packets/image (SF7, 85B payload)	Not Specified	Not Specified	SX1276/SX1278	Not Specified	Greece (Rhodes Island)
[11]	Cai et al.	OV2640 (640 × 480)	ESP32 + CubeCell-AB02 (LoRa) + WM1302 + RPi	Np = 20 packets/image, 140 packets/day	Avg current = 507 mA; 581 days battery life (10,000 mAh)	SX1302 (WM1302 module)	Not Specified	Not Specified	Hong Kong (Tsuen Wan)
